# A novel rat CVB1-VP1 monoclonal antibody 3A6 detects a broad range of enteroviruses

**DOI:** 10.1038/s41598-017-18495-4

**Published:** 2018-01-08

**Authors:** Niila V. V. Saarinen, Jutta E. Laiho, Sarah J. Richardson, Marie Zeissler, Virginia M. Stone, Varpu Marjomäki, Tino Kantoluoto, Marc S. Horwitz, Amirbabak Sioofy-Khojine, Anni Honkimaa, Minna M. Hankaniemi, Malin Flodström-Tullberg, Heikki Hyöty, Vesa P. Hytönen, Olli H. Laitinen

**Affiliations:** 1 Faculty of Medicine and Life Sciences, University of Tampere, Tampere, Finland; 2 University of Exeter Medical School, Exeter, Devon, UK; 3 Department of Medicine HS, Karolinska Institutet, Stockholm, Sweden; 4 Fimlab Laboratories, Pirkanmaa Hospital District, Tampere, Finland; 5 Department of Biological and Environmental Science/Nanoscience center, University of Jyväskylä, Jyväskylä, Finland; 6 Department of Microbiology and Immunology, Life Sciences Institute, University of British Columbia, Vancouver, Canada

## Abstract

*Enteroviruses* (*EVs*) are common RNA viruses that cause diseases ranging from rash to paralytic poliomyelitis. For example, *EV-A* and *EV-C* viruses cause hand-foot and mouth disease and *EV-B* viruses cause encephalitis and myocarditis, which can result in severe morbidity and mortality. While new vaccines and treatments for EVs are under development, methods for studying and diagnosing EV infections are still limited and therefore new diagnostic tools are required. Our aim was to produce and characterize new antibodies that work in multiple applications and detect EVs in tissues and *in vitro*. Rats were immunized with Coxsackievirus B1 capsid protein VP1 and hybridomas were produced. Hybridoma clones were selected based on their reactivity in different immunoassays. The most promising clone, 3A6, was characterized and it performed well in multiple techniques including ELISA, immunoelectron microscopy, immunocyto- and histochemistry and in Western blotting, detecting EVs in infected cells and tissues. It recognized several *EV-Bs* and also the *EV-C* representative Poliovirus 3, making it a broad-spectrum EV specific antibody. The 3A6 rat monoclonal antibody can help to overcome some of the challenges faced with commonly used EV antibodies: it enables simultaneous use of mouse-derived antibodies in double staining and it is useful in murine models.

## Introduction

*Enteroviruses* (*EVs*) belong to the family of Picornaviridae and are small non-enveloped viruses with a single-stranded positive-sense RNA genome. There are thirteen enterovirus species, seven of which affect humans. Currently, nearly 300 human EV serotypes/genotypes, including Rhinoviruses have been identified (http://www.picornaviridae.com/enterovirus/enterovirus.htm, May 30^th^ 2017). The majority of EV infections cause only mild common cold-type symptoms, or are asymptomatic, however, in some cases they can trigger more severe acute and chronic diseases such as myocarditis, chronic cardiomyopathy, hand, foot and mouth disease, paralysis, encephalitis and meningitis^1,2^. In some clinical forms the main contributing EVs that cause a particular disease are well known. Three poliovirus serotypes cause poliomyelitis and Coxsackievirus B (CVB) serotypes are the main cause of acute viral myocarditis^1^. Correspondingly, EV71 and EV68 have recently been found to cause severe neural symptoms^3,4^. However, in the case of other diseases like type 1 diabetes (T1D), where a link between EVs and the onset of the disease has been proposed and several candidate serotypes have been identified^5–8^ the association has not been fully established. Therefore, it is essential to develop tools and techniques that are capable of recognizing multiple EV serotypes for detecting the range of EVs that could potentially contribute to these different diseases.

The EV capsid is an icosahedron that consists of repeats of four structural proteins, VP1-4. VP1, VP2, and VP3 are located on the outer surface of the capsid, whereas VP4 resides on the inner capsid surface^9^. The EV VP1 protein displays the greatest sequence variation between serotypes, but it also contains a conserved immunodominant epitope^10,11^. Therefore, it is an attractive candidate for the development of diagnostic tools to both detect EVs and differentiate between EV groups. There are a number of different EV antibodies available, which vary in their species and serotype specificity, such as Cox mAB 31A2 that binds to certain CVB types^12^ (Laiho *et al*. submitted Virchows Archiv 2017) and others that react more widely with different EV types, such as the monoclonal antibody 5D8/1^13^ by Dako Cytomations, Denmark (referred hereafter as 5D8/1) and the 9D5 (Millipore); the last two are widely used in EV research^14^. These antibodies each have their own strengths and shortcomings with regards to sensitivity and specificity^12,14,15^. For example, the cross-reactivity of 5D8/1 to smooth muscle tissue is well documented^12,15^, which may make the interpretation of results more challenging in tissues like heart and intestine. The Cox mAB 31A2, which was raised against the VP1 of CVB3, recognizes CVB1 and CVB3^12^ (Laiho *et al*., submitted), but not other EV serotypes. This narrow recognition pattern of 31A2 needs to be considered when it is used in clinical diagnostics.

Mice are the most commonly used animal model for studying EV infections and pathology^16,17^ and due to the fact that most monoclonal antibodies against EVs are based on mouse hybridomas, their use in histology can be problematic due to high background caused by endogenous antibodies (mouse-on-mouse unspecific binding)^18^. Therefore, there is a need for novel non-mouse EV antibodies. To address this, we generated a new rat monoclonal EV antibody, clone 3A6, that solves the host-species issue and that can help to overcome some of the current challenges associated with other EV-antibodies in research and diagnostics.

## Objectives

Our aim was to develop a novel monoclonal antibody that recognizes an EV group-specific epitope located in the N-terminus of VP1 protein and that could be used in multiple applications to study EVs. Rats were chosen for the generation of the hybridoma clones to address the challenges faced with mouse-derived monoclonal antibodies which are currently widely utilized in EV research.

## Results

### 3A6 recognizes CVB viruses and shows reactivity towards VP1 N-terminus in ELISA

**Figure 1:**
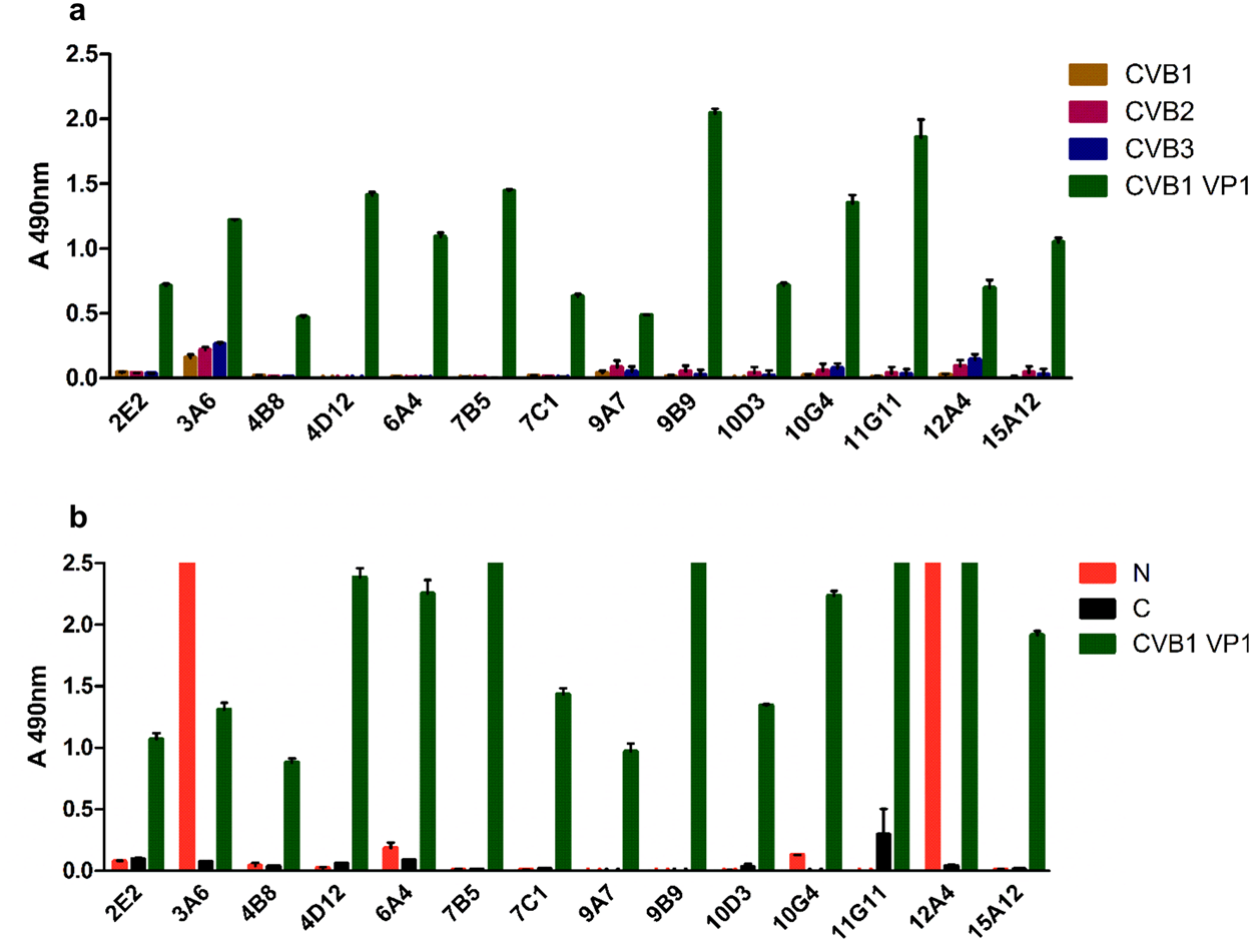
Reactivity of 14 different monoclonal CVB1-VP1 antibody clones against CVB1-3 viruses and CVB1 VP1 as well as mapping of their binding region in ELISA. (**a**) Screening of hybridoma supernatants against purified CVB1-3 viruses and CVB1 VP1 protein. Virus samples were heat-inactivated before coating. (**b**) Screening of hybridoma supernatants against CVB1 VP1, and the N- and C-terminal fragments of CVB3 VP1. Samples were run as duplicates, with 250 ng/well of antigen. The absorbance of blocked wells was subtracted from the sample absorbance values before plotting the data. Error bars show standard deviation of duplicates. In (**b**) N and C refers to terminal GST fusion constructs. Signal intensity differences between (**a**) and (**b**) are due to differing incubation times with substrate.

**Figure 2:**
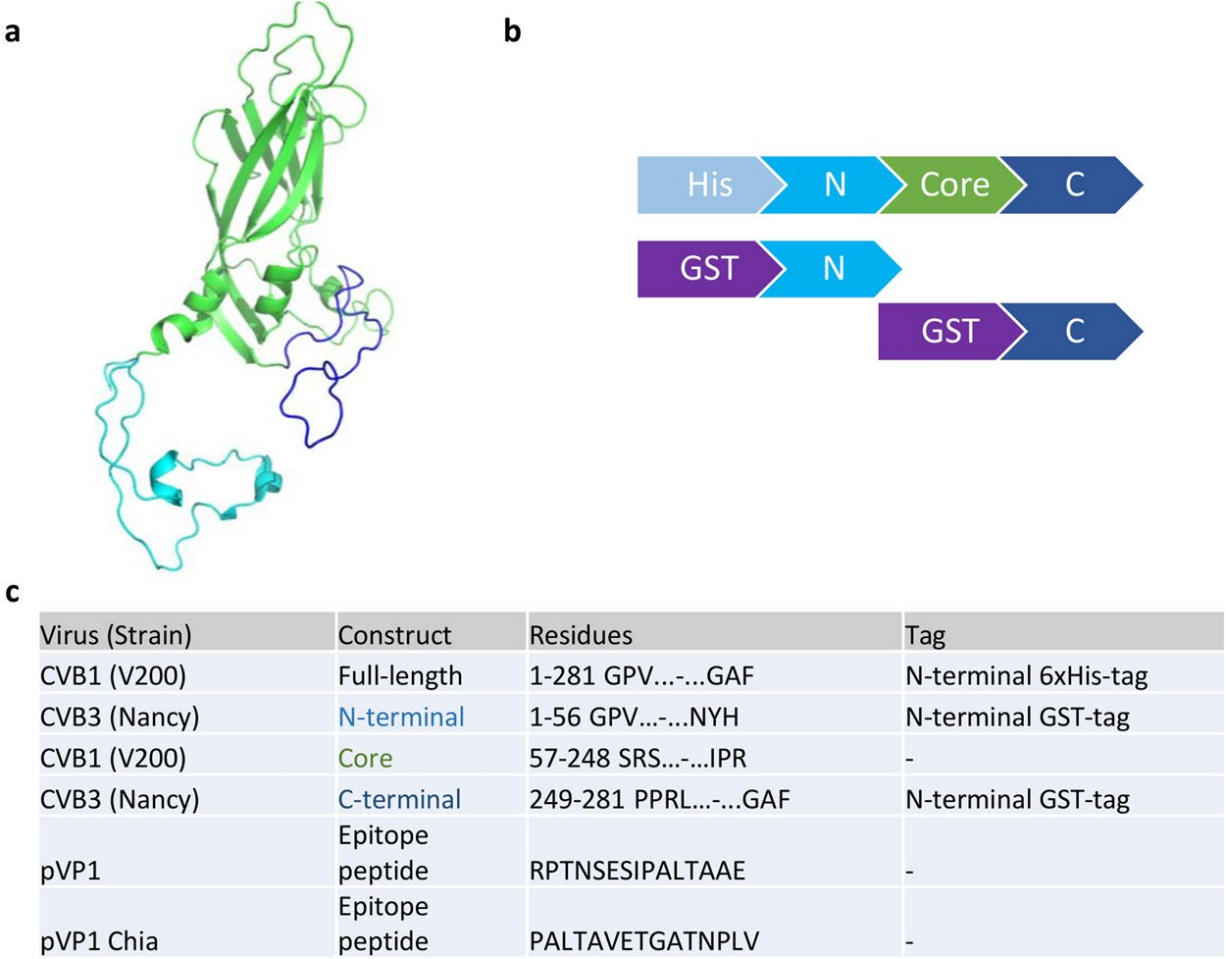
Design of the VP1 constructs used in antibody generation and ELISA screening. (**a**) CVB3 VP1 structure (PDB id 4GB3) presented as cartoon model showing the N-terminal segment (light blue), the core region (green) and the C-terminal part of the protein (dark blue). (**b**) Schematic illustrations of full length CVB1 VP1 and truncated CVB3 VP1 expression constructs used for immunization and for immunoassays. (**c**) Recombinant protein constructs and peptides used in the antibody generation (full-length CVB1 VP1) and in ELISA assays (all constructs except Core region).

All 14 generated monoclonal antibodies were strongly positive for CVB1 full-length VP1, but binding to heat inactivated viruses was detectable only with clones 3A6 and 12A4 (Fig. 1a). Based on the preliminary screening of the clones using ELISA and immunocytochemistry (data not shown), five clones were selected for further studies: two staining/binding positively to the N-terminus of CVB3 VP1 (clones 3A6 and 12A4) and three binding other parts of VP1 (4D12, 7C1, 9B9) (Fig. 1). For the general structure of VP1 protein and its parts, see Fig. 2a and b. Interestingly, none of the clones were strongly binding the VP1 C-terminus (Fig. 1b). Out of these clones, 3A6 showed the highest potential as an antibody with broad reactivity against EVs as it gave the strongest signal when purified viruses were used as antigens (Fig. 1a). Therefore, we focused our further efforts in characterizing 3A6.

### The 3A6 binding site on the N-terminus of VP1 overlaps with the enterovirus group specific epitope

**Figure 3:**
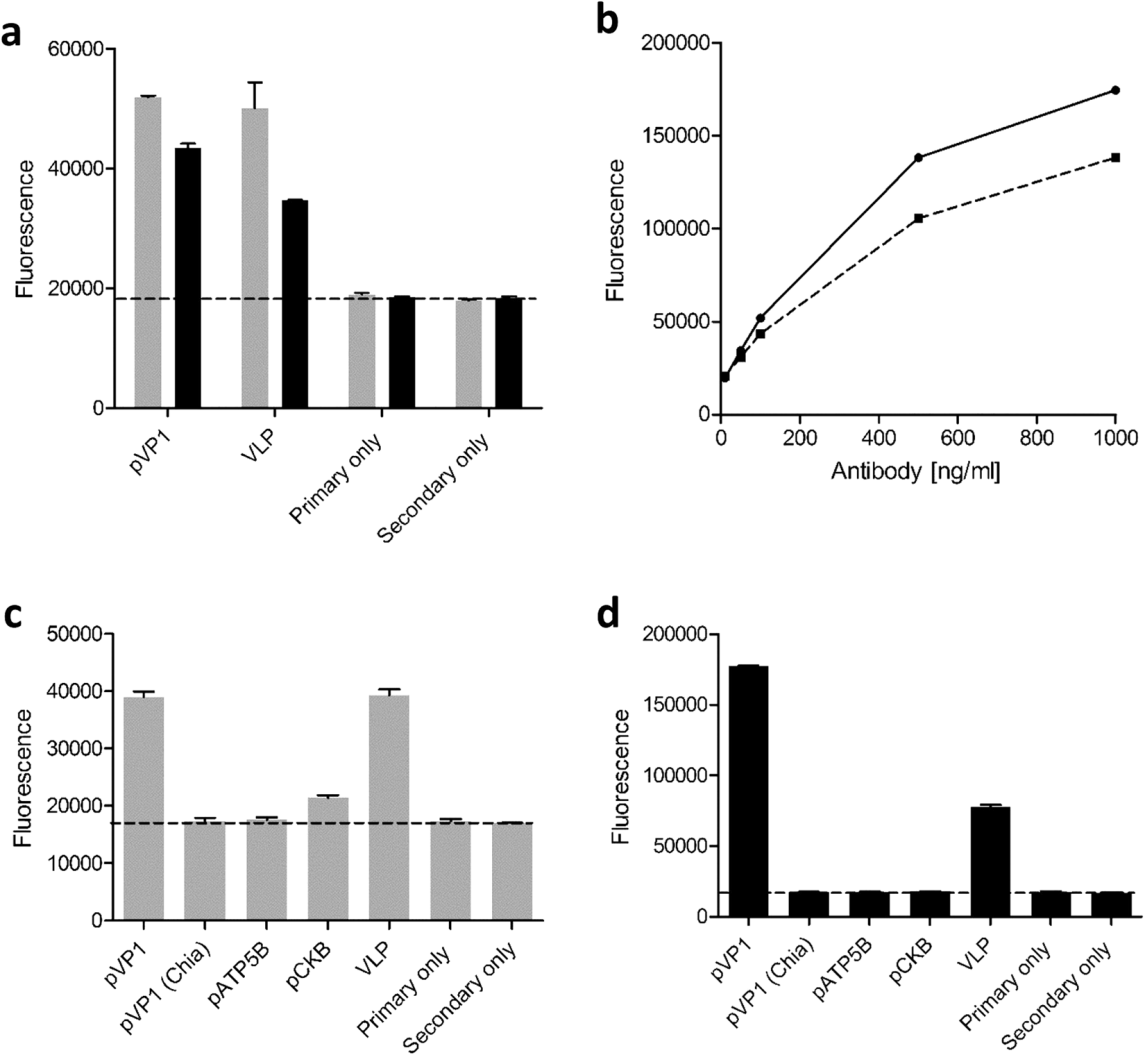
Binding of 3A6 and 5D8/1 antibodies to different antigens in immunofluorescence assay. In (**a**) the binding of 5D8/1 (grey bars) and 3A6 (black bars) to pVP1 peptide RPTNSESIPALTAAE (500 ng/well) and CVB3 VLP (250 ng/well) was compared. (**b**) A dose response curve of 5D8/1(black line) and 3A6 (dotted line) binding to VP1. (**c**) The binding of 5D8/1 and 3A6 (**d**) to peptides derived from VP1 (VP1 – RPTNSESIPALTAAE; VP1 Chia – PALTAVETGATNPLV), CKB, ATP5B and whole virus capsid representing CVB3 VLP.

As we identified that the epitope of 3A6 resides in the N-terminal region of VP1 (Fig. 1) and as it is known that the 5D8/1 epitope also locates in the N-terminal part of VP1^10^, we examined whether 3A6 binds to the same or to a unique epitope. To study this, two peptides (Fig. 2c) previously used to characterize the epitope bound by 5D8/1^19^ were utilized in an Immunofluorescent assay (IFA). We used CVB3 VLP as a positive control for binding^20^. Peptide IFAs confirmed that both the 3A6 and the 5D8/1 antibodies recognize the VP1 peptide RPTNSESIPALTAAE (Fig. 3a–d), however neither antibody recognized the overlapping downstream peptide PALTAVETGATNPLV (Fig. 3c and d), suggesting that the 3A6 epitope overlaps with that of 5D8/1, and if they are not the same, the epitope bound by 3A6 may be located closer to the N-terminus of VP1. We also studied whether either antibody could bind to peptide epitopes of CKB or ATP5B (Fig. 3c and d), two mitochondrial proteins proposed to cross-react with 5D8/1^15,19^. We observed a weak binding of 5D8/1 to the CKB peptide (Fig. 3c), whereas 3A6 showed no reactivity towards these two peptides (Fig. 3d).

### 3A6 recognizes CVB1-6 in Western blotting with similar intensity

**Figure 4:**
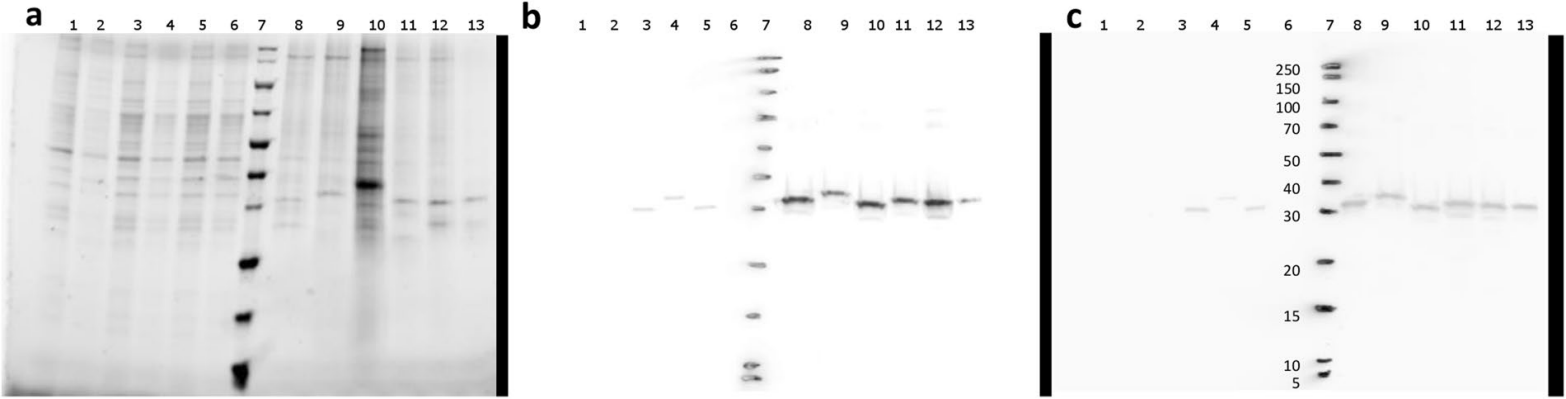
Comparison of 3A6 and 5D8/1 enterovirus detection profiles in Western blotting. (**a**) Sample total proteins visualized with the Stain-Free technology. (**b**) Immunoblot stained with 5D8/1 (1:3000) and (**c**) with 3A6 (1:1000). VP1 proteins are ~30 kDa, depending on the EV serotype. Sample order from left to right: lysates from non-infected GMK and Vero cells (8 µg/lane; lanes 1 and 2), CVA9, Echovirus 6, Echovirus 30 and Poliovirus 3 (Sabin) infected cell lysates (8 µg/lane; lanes 3–6), ladder (lane 7) and concentrated and quantified CVB1–6 (0,66 µg) (lanes 8–13).

In order to compare how 3A6 and 5D8/1 antibodies detect different EVs in Western blotting, EV-infected cell lysates and concentrated CVB viruses were used. Both antibodies detected CVB serotypes 1-6, Echovirus 6, Echovirus 30 and Coxsackievirus A9 in infected cell lysates, but neither of them stained Poliovirus 3 (PV3). PV3 VP1 has a lysine at position 40, where in *EV-B* there is an uncharged amino acid, usually glutamine (Supplementary Figure S1). This might explain the lack of binding in WB, where the epitope is linearized when compared to IHCP and IF. Neither antibody gave unspecific binding to non-infected cells or proteins in extracts of infected cells (Fig. 4: lanes 1–2, 3–6 and 8–13). Unlike 5D8/1, 3A6 showed less variation in signal intensity between the different CVB serotypes (Fig. 4: lanes 8–13) particularly when comparing their relative binding to CVB5 and CVB6 (Fig. 4; lanes 12 and 13 in panels b and c).

### 3A6 recognizes EV-infected cells in immunocytochemistry (ICC)

The ability of 3A6 and 5D8/1 to recognize different EV serotypes was further tested in chromogenic ICC and in IFA by two independent laboratories (Tampere, Finland and Exeter, UK) using an array of EV-infected cells (Table 1). Both antibodies recognized the *EV-B* viruses well, but only 5D8/1 showed some reactivity towards viruses from *EV-A* species. In addition, both antibodies recognized PV3, which belongs to *EV-C*. Neither of the antibodies bound the non-infected control cell lines (A549, GMK, Vero, RD and HeLa), or the Adenovirus or Human parechovirus 1 (HPeV1) infected cells (Table 1). A slight variation was observed in the staining intensity between IF and IHC-P (Supplementary Figure S2) however, the overall recognition spectrum was similar in both assays. The 3A6 also recognized all tested CVB1 (N = 30) strains in the CVB1 cell microarray^21^.

**Table 1:** 3A6 mainly recognizes EVs of the B species. A cell microarray including 20 different EVs and two non-EV viruses (Adenovirus C and Human Parechovirus 1) was immunostained with 3A6 in two independent laboratories to verify the recognition spectrum of the antibody. The results of chromogenic immunostaining by 3A6 are shown in comparison to those obtained with 5D8/1^21,24^ are also shown. Recognition scale: +++ = strong, ++ = moderate, + = weak, − = negative.

Virus			Cell line	Antibody	
Species	Serotype	Strain		Rat anti-VP1 mAb	Mouse anti-VP1 mAb
				Clone 3A6	Clone 5D8/1
EV B	CVB1	ATCC	GMK	++	+++
EV B	CVB2	ATCC	GMK	++	+++
EV B	CVB3	ATCC	GMK	++	+++
EV B	CVB4	ATCC	GMK	++	+++
EV B	CVB5	ATCC	GMK	+++	+++
EV B	CVB6	ATCC	GMK	++	++
EV B	Echo3	PB-E3DiT23	GMK	+	+
EV B	Echo4	ATCC	GMK	++	+
EV B	Echo6	ATCC	GMK	+++	+++
EV B	Echo9	ATCC	GMK	++	++
EV B	Echo11	ATCC	GMK	+++	+
EV B	Echo30	ATCC	A549	+++	+++
EV B	CVA9	ATCC	GMK	++	+++
EV B	CVA9	PB-CVA9V59	RD	++	++
EV A	CVA2	PB-CVA2V38	RD	−	+
EV A	CVA4	PB-CVA4V36	RD	−	+
EV A	CVA5	PB-CVA5V43	RD	−	++
EV A	CVA6	PB-CVA6V303V	RD	—	+
EV A	CVA10	PB-CVA10V2530	RD	—	+
EV A	CVA16	PB-CVA16V55	RD	—	+
EV A	CVA16	ATCC	Vero	—	—
EV A	EV71	PB-EV71Hus	GMK	—	—
EV C	PV3	Sabin	GMK	++	++
Adeno	C	VR846	HeLa	−	−
HPeV	HPeV1	ATCC	A549	−	−

**Figure 5:**
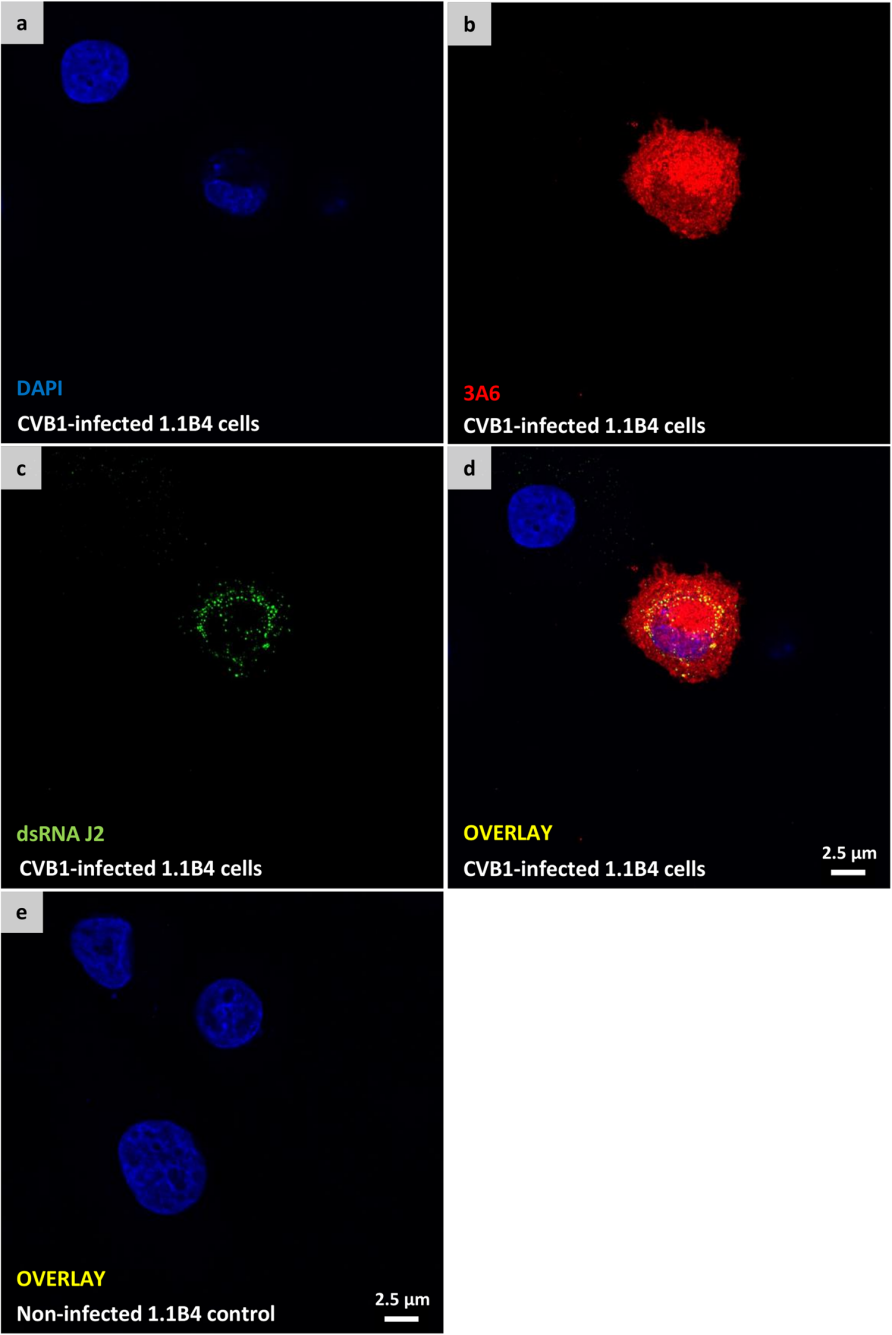
3A6 recognizes enterovirus in persistently infected 1.1B4 cells. Confocal images of PFA-fixed chronically CVB1-infected 1.1B4 cells (**a**–**d**) and non-infected 1.1B4 control cells (**e**) double stained with 3A6 (red) and dsRNA antibody J2 (green). Merged images of the double stained infected- and non-infected cells are visualized in (**d** and **e**), respectively.

To test the suitability of 3A6 to recognize both acutely-infected and chronically-infected cells in non-paraffin embedded PFA-fixed coverslips, samples with acutely CVB1-infected PANC-1 cells and chronically CVB1-infected 1.1B4 cells (unpublished model that is based on previously published data^22^) were utilized. The coverslips were co-stained with 3A6 and dsRNA antibody^23^ J2 to visualize the localization of viral protein and dsRNA in the same cell. Both antibodies worked well under the conditions tested. 3A6 staining was distributed throughout the cell cytosol whereas J2 had punctate pattern (Fig. 5, Supplementary Figure S3). In addition, dual staining with 3A6 and 5D8/1 in CVB1-infected Vero cells showed that the two antibodies co-localized (Supplementary Figure S4).

The relative sensitivity of 3A6 in other EV detection methods was tested with IFA and IHC-P staining using the previously published limited dilution series cell microarray (CMA)^24^, which involves dilutions of A549 cells infected with CVB1. In both assays the antibody detected virus infections in cells diluted to 10^−3^, reflecting a sensitivity similar to that as previously obtained with several polyclonal EV antibodies^24^. However the 3A6 showed lower sensitivity when compared to 5D8/1 in IHC-P (10^−6^)^24^.

### 3A6 detects acute CVB infections in tissue samples by immunohistochemistry

**Figure 6:**
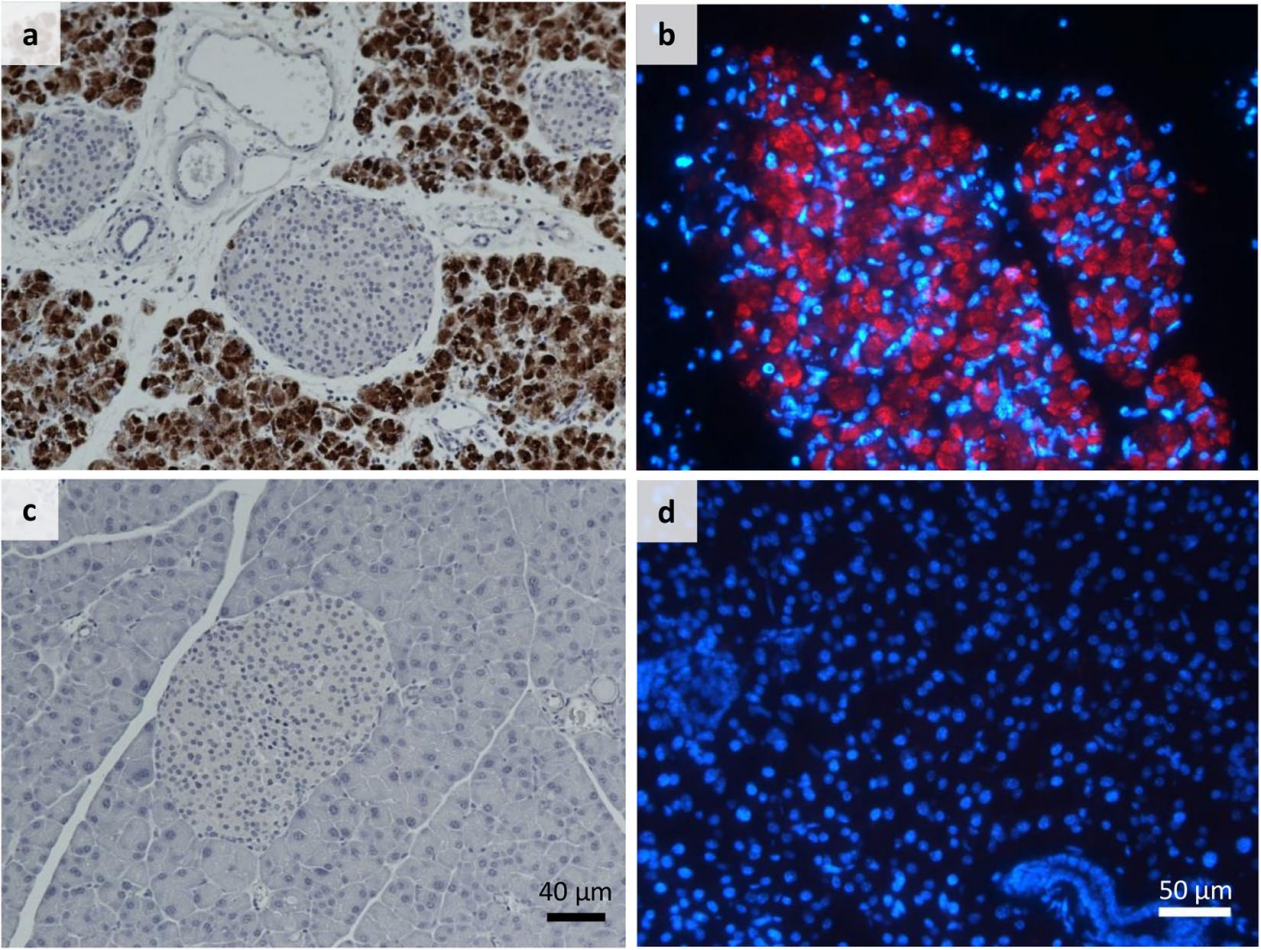
3A6 recognizes CVBs in infected mouse pancreata. CVB1- and CVB3-infected mouse pancreata were stained with 3A6 by IHC-P and IF (**a** and **b**), respectively. Positive signal from the virus recognition is indicated by brown (**a**) and red colors (**b**). The non-infected mouse pancreata (**c**) and (**d**) were used as negative controls. (**a**,**c** and **b**,**d**) have the same magnification. Blue color in (**b**) and (**d**) is DAPI, which stains the cell nuclei.

To study the applicability of 3A6 in IHC, we tested it in different mouse and human tissue samples. In EV-infected mouse pancreas, 3A6 showed a clear reactivity towards CVB1 and CVB3 (Fig. 6), and CVB4 (not shown), with no background staining in non-infected mouse pancreata (Fig. 6c and d). As shown previously^25^ the CVBs preferentially target the exocrine tissue in the mouse pancreas.

**Figure 7:**
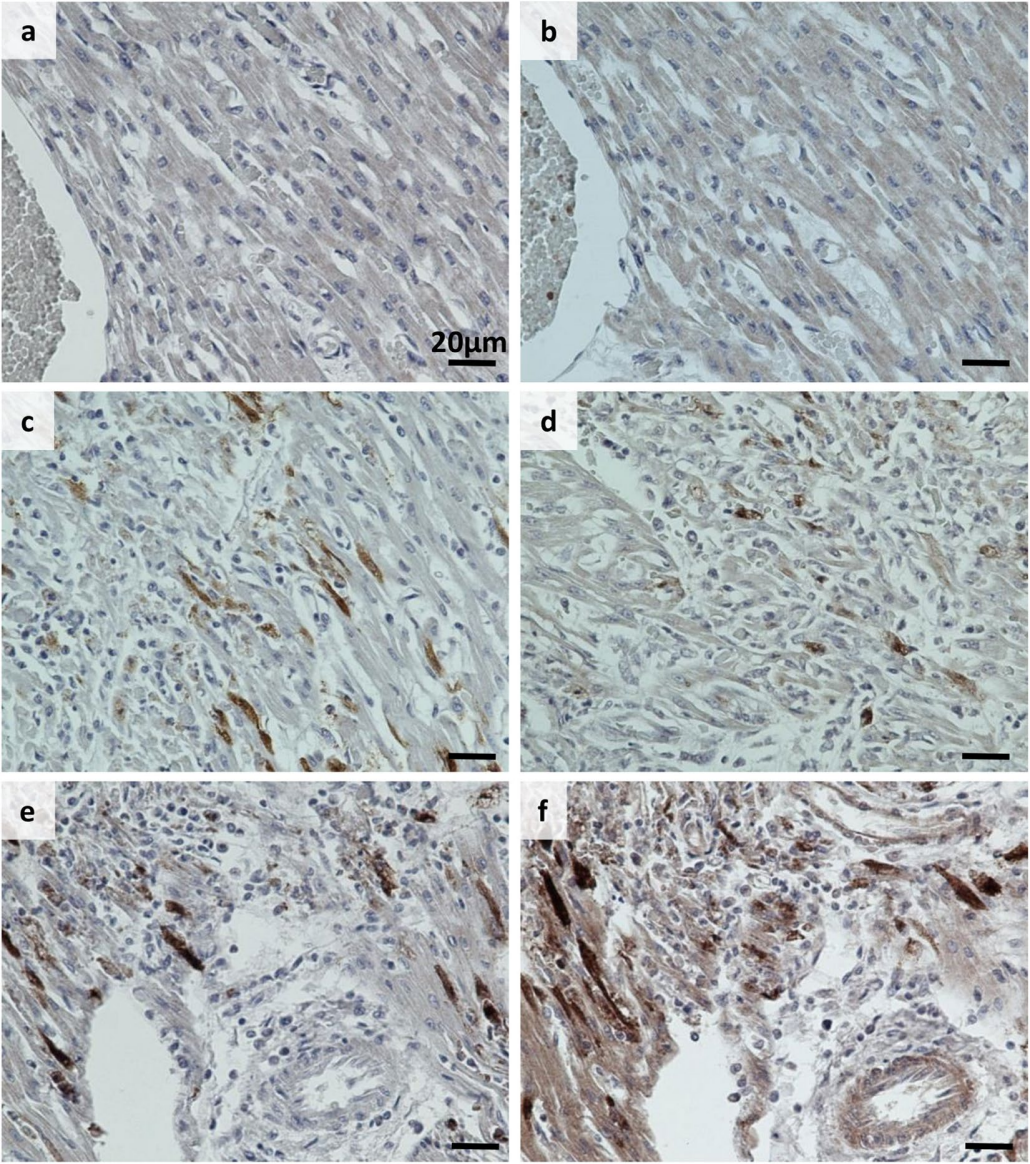
Comparison of 3A6 and 5D8/1 detection of virus in neonate heart tissue with IHC-P. Representative images of Clone 5D8/1 (**a**,**c** and **e**) and 3A6 (1:700) (**b**,**d** and **f**) staining in control heart (**a** and **b**) and heart from neonates with either a lethal culture-confirmed CVB2 infection (**c** and **d**) or CVB4 infection (**e** and **f**). Scale bar 20 μm.

Human pancreas and spleen samples from the PanFin network^26^ with unknown EV status were stained with 3A6, 5D8/1 and rat isotype antibody control in consecutive sections. These samples showed no EV positivity with these antibodies and the rat isotype control was also negative. Furthermore, heart tissue from a confirmed CVB infection was stained with 3A6 and 5D8/1: clear staining was observed with both antibodies in the infected tissue, but not in non-infected control heart (Fig. 7).

### 3A6 labels VP1-proteins in cryo-immuno EM

**Figure 8:**
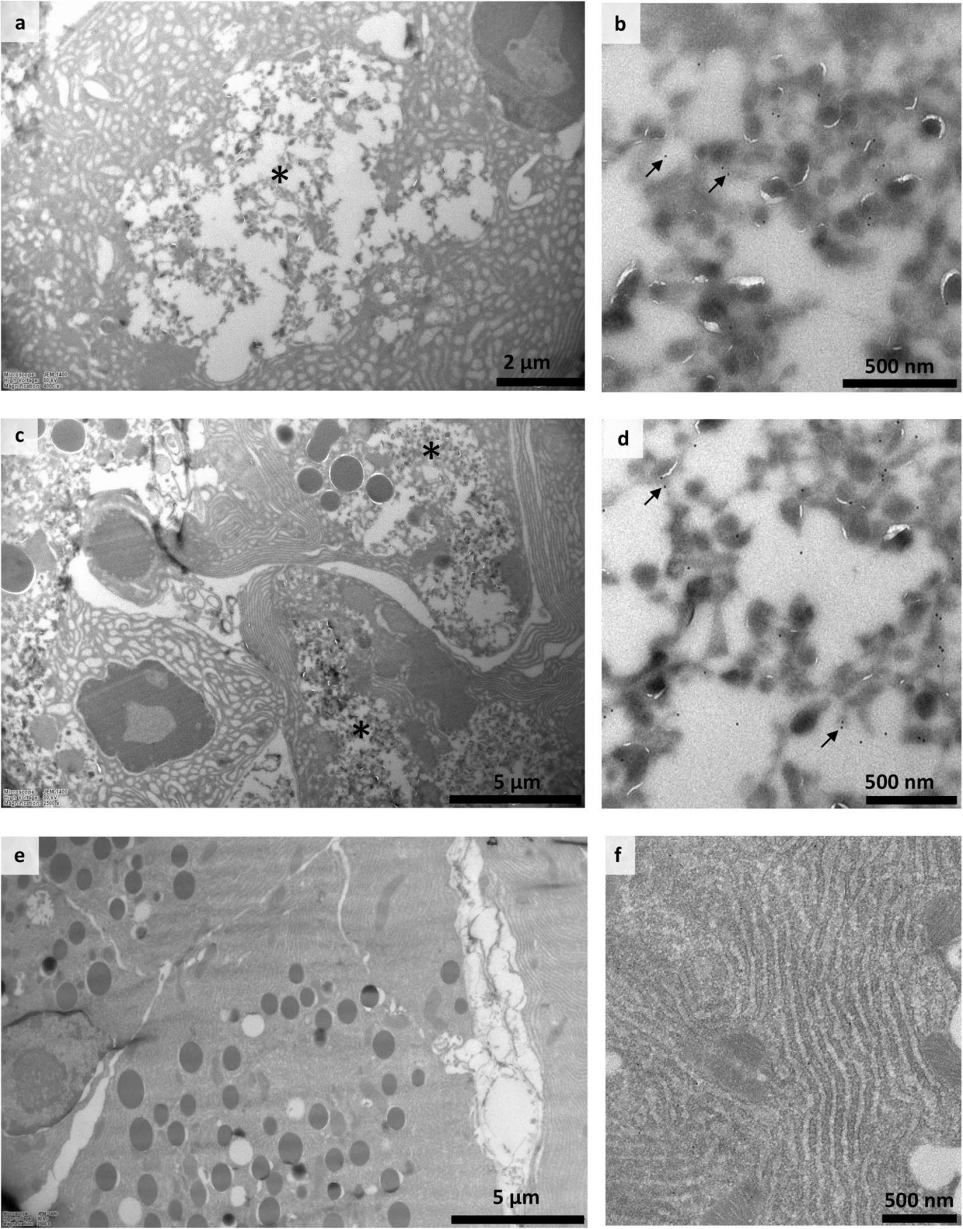
Immunolabeling of CVB1 infected mouse tissue in cryo-immuno EM with 3A6 and 5D8/**1**. Sections from CVB1 infected (**a**–**d**) and non-infected (**e**,**f**) mouse pancreas exocrine tissue acinar cells three days post infection: labelled with antibodies 5D8/1 (panels a, b and e) and 3A6 (panels c, d and f). Infected tissues (**a,c**) show gross changes during infection, and panels on the right (**b,d**) show details of cytoplasmic membranous structures positive for CVB1 VP1. Dots indicated by arrows are protein A gold labeled antibodies. Areas of novel membranous structures formed during infection are indicated by asterisks (**a,c**).

Cryo-immuno-EM was used to study the VP1 containing cellular compartments in CVB1 infected exocrine pancreatic cells using both 3A6 and 5-D8/1 antibodies. Mouse pancreas tissue showed clear signs of infection 3 days after intraperitoneal inoculation with CVB1 (10^5^ PFU/mouse) (Fig. 8). The presence of virus in the pancreas was also confirmed by PCR, which showed high levels of CVB1 (data not shown). At low magnification, morphological changes due to infection were obvious: apoptotic nuclei, swollen endoplasmic reticulum, emergence of novel membranous structures in the cytoplasm. These tubulovesicular structures, which are characteristic for infected cells^27^, were antibody positive (3A6 and 5D8/1) and not detected in non-infected cells.

### 3A6 does not neutralize CVB1

No neutralizing activity of CVB1 was observed with any of the 14 culture hybridoma supernatants. Also, no neutralization activity was seen in the total antisera of the rats immunized with VP1.

## Discussion

EV infections are common, especially in children, and their clinical manifestations range from subclinical to severe or even fatal outcomes. Most EV infections are mild, but tools are required for detecting EVs from clinical samples, including blood, cerebral spinal fluid, stool and in histologic sections in more serious infections. As multiple EV types are linked to diseases like viral myocarditis, encephalitis and type 1 diabetes^1,28^, reagents that are capable of detecting a broad range of different EV types with both high specificity and sensitivity are needed.

Recently, the broad-spectrum EV antibody 5D8/1 from Dako has been regularly used in both clinical and research settings. This antibody is a mouse monoclonal that recognizes an epitope within the capsid protein VP1^11,19^. 5D8/1 mainly identifies *EV-B* viruses, including CVBs and echoviruses, but also many species A EVs and poliovirus 3 of the C species. However, in non-optimal conditions 5D8/1 has been reported to cross-react with some cellular proteins such as CKB and ATP5B^12,15^. As such, there is an ongoing effort to produce better antibodies with superior selectivity and specificity due to the limit in the number of specific antibodies that recognize a broad-range of EVs. With this aim, the new broad-reactive rat monoclonal antibody 3A6 was generated. 3A6 detects all CVBs, along with other *EV-Bs* (Table 1 and Fig. 4). In addition to broad-reactivity, we aimed to produce an antibody that will further support the findings made with other EV antibodies, and improve detection in different experimental models, including the mouse.

The 3A6 antibody was successfully validated for use in different methods including Western blotting, peptide IFA, immuno-TEM, IHC-p and IFA. IHC-P and IFA methods were tested for paraffin and PFA-fixed samples of different origins. 3A6 worked well under the acute infection settings, both *in vitro* and *in vivo* derived models as well as with a persistent infection *in vitro* model. As a rat antibody, the 3A6 will be especially advantageous in mouse models, since no cross-reactivity to mouse cellular proteins was observed. We also tested 3A6 alongside 5D8/1 in human tissue samples, some of which had been previously confirmed positive for EVs^1^. In human samples 3A6 did not show non-specific binding in non-infected tissues and showed a comparable signal to 5D8/1 in infected tissues (Fig. 7). 5D8/1 showed reactivity to smooth muscle that was not seen with 3A6 (not shown). We also observed that antigen retrieval at pH 9 using Tris-buffer resulted in non-specific binding of 3A6 to red blood cells in some cases, but this could be avoided by using citrate buffer at pH 6.

We showed that 3A6 and 5D8/1 co-stain the same cellular areas (Supplementary Figure S4), which renders 3A6 as a valid diagnostic tool replicating the results achieved with 5D8/1. For instance: double staining with 3A6 and 5D8/1 tissues in IFA could help validate incoherent findings as true positives or negatives for example in sections with smooth muscle tissue.

There are relatively few studies showing the high-detail morphological changes occurring in EV infections^27^. Here, we show several changes related to infection and replication of EVs in the cytoplasm of acinar pancreatic cells, including nuclear apoptosis, endoplasmic reticulum swelling and novel membranous structures in the cytoplasm containing CVB1 VP1 evidenced with either 3A6 or 5D8/1. Importantly, we also show that 3A6 can be used in combination with the dsRNA mouse antibody J2 to simultaneously visualize the localization of viral protein and replicating RNA within infected cells (Fig. 5 and Supplementary Figure S3). These results together demonstrate that 3A6 can be used for studying the intracellular localization of the viruses.

In Western blotting, the 3A6 recognizes the six CVB serotypes with similar sensitivity, whereas CVB6 detection with 5D8/1 could have been missed, with a shorter exposure time (Fig. 4b and c). Thus, the conditions in some assays, such as Western blotting, seem to favor 3A6. Additionally, 3A6 has already proven to be very useful in our own quality controls when culturing and purifying enteroviruses^29^ and their VP1 proteins (Saarinen, unpublished).

Although 5D8/1 is a broadly reactive antibody, it cannot neutralize CVB1 infections *in vitro* ^13^. Also 9D5 lacks neutralizing capacity, which is understandable since its epitope, just like that in 5D8/1, is not exposed in an intact virus particle^14^. Similarly, none of our 14 individual monoclonal antibodies targeting VP1, or polyclonal sera from VP1-immunized rats, could neutralize CVBs. This indicates that recombinant proteins which do not represent the native EV capsid may not be efficient in the generation of antibodies capable of blocking viral infection. The above observations question the rationale of VP1-based subunit vaccines for preventing EV infections^28^.

As the number of broadly-reactive and serotype-specific EV antibodies expands, the construction of a panel of antibodies that overlap in their ability to bind different EVs the detection of group and serotype specific EV infections will improve. Maccari *et al*.^14^ suggested that simultaneous use of 5D8/1 and 9D5 allows for the identification of enterovirus groups based on their differences in their ability to bind to different EVs. Adding 3A6 to this panel, which does not bind to *EV-A* viruses would make such a test more accurate. Importantly, binding differences could be utilized to construct a multiplexing application based on biosensors to identify a wide range of EV serotypes from liquid samples.

In conclusion, the new rat 3A6 antibody is a versatile tool that can be used in multiple applications. It has been tested in three laboratories with consistent results and shows no unspecific binding.

### Availability of data and material

The Finnish Funding Agency for Innovation (Tekes) has funded this research. According to their funding terms Finnish companies involved in the project have a first right of refusal to the IPR generated in the research project. Therefore, we cannot reveal details that could be covered by these terms such as sequences of antibodies. We are, however, willing to provide samples of 3A6 antibody to the scientific community worldwide free of charge.

## Materials and Methods

### Tissues used in the study

The mouse tissue sections were cut from paraffin embedded pancreas tissue described in^21^. The tissue was derived from non-obese diabetic (NOD) mice infected with CVB1, CVB3, or buffer alone. The mouse tissue used in Cryo-immuno Electron microscopy were from University of British Columbia where all animal work was performed under strict accordance with the regulations of the Canadian Council for Animal Care. The protocol was approved by the Animal Care Committee of the University of British Columbia (certificate number A13-0116). The paraffin embedded human tissue samples used in this study were from the UK collection^30^ or from the PanFin network^26^. Handling of rats was performed by Genscript.

### Generation of VP1 expression constructs, expression and purification of VP1 proteins

DNA encoding for CVB1 (GenBank: EU147493.1) VP1 with an N-terminal His-tag (Fig. 2b and c) and flanking AttL-sites was obtained from Gene Art (ThermoFisher). The construct was cloned into pBVboostFGII + WPRE-vector^31^ utilizing Gateway® LR Clonase® enzyme mix (ThermoFisher Scientific). BL21 Star™ (DE3) One Shot® *E*. *coli* (Invitrogen) were used to produce the recombinant VP1 protein in a 7.5 l bioreactor (Infors-HT3; Labfors) using the fed batch protocol utilizing pO2-stat as described in^32^. Bacteria were pelleted by centrifugation, resuspended in binding buffer (20 mM phosphate buffered, 20 mM imidazole, 500 mM NaCl; pH7.4) and subsequently lysed using the Emulsiflex C3 homogenizer (Avestin). The lysate was clarified by centrifugation at 20, 000 g for 20 min at + 4 °C and the recombinant VP1 was purified with immobilized metal anion chromatography using 5 ml TALON Superflow Co-NTA columns (GE Healthcare) with Äkta Purifier (GE Healthcare). Purified VP1 protein was detected by immunoblot using the primary antibody 5D8/1 (Dako Cytomations) followed by peroxidase labeled horse anti-mouse IgG (H + L) antibody (Vector Laboratories Inc). Purified protein was dialyzed into phosphate buffered saline (PBS) and subsequently sent to Genscript for antibody generation.

Constructs with either the N-, or C-terminal regions of CVB3 VP1 (GenBank: M33854.1) fused with Glutathione S-transferase (GST) (Fig. 2b and c) were ordered as synthetic genes (Genscript) and subcloned into pGEX-2T. The terminal fragments were produced as described above and the purification was carried out using Protino® Glutathione Agarose 4B resin with gravity flow using 100 mM Tris-HCl pH 7.5 + 150 mM NaCl as a binding buffer. Proteins were eluted with 100 mM Tris-HCl pH 7.5 + 150 mM NaCl + 20 mM glutathione and dialyzed into TBS.

### Generation of rat derived monoclonal antibody clones

Genscript performed the rat immunizations: five rats were injected thrice with 50 µg recombinant CVB1 VP1 in PBS. After 90 days, the animals were sacrificed and their spleen cells were electrofused with rat carcinoma cells. Genscript also performed the prescreening of animal serum and hybridoma supernatants against CVB1 VP1 and CVB3 N-terminal region of VP1, along with counter-screening against their in house his-tag control. Supernatants from 14 hybridoma clones were further tested in Tampere with ELISA and Western Blotting using different parts of the VP1 protein as antigens, as well as in immunofluorescence (IF) to screen for different EVs in cell samples infected with known viruses.

### ELISA screening of monoclonal antibodies

Supernatants of hybridoma cell cultures were used to screening candidate clones. CVB1-3, purified from respective field isolates^29,33^ and VP1 proteins (Fig. 2) were used as antigen to coat Maxisorp™ (Nunc) wells (250 ng/well). Coating was performed in 50 mM carbonate buffer pH 9.6 at RT overnight with 0.1% BSA blocking. The supernatants and the secondary antibodies were diluted in TBS containing 0.05% Tween 20 (Sigma) and 1% BSA (BSA-TBST). HRP-conjugated Goat anti Rat IgG (H + L) (Life technologies™) was used as a secondary antibody (1:1000) in BSA-TBST, and O-nitrophenyl diamine (Sigma) was used for detection. Absorbance was measured at 590 nm using a Perkin Elmer Wallac 1420 Victor2 microplate reader. The approximate location of the antibody epitopes was probed using the full-length CVB1 VP1 and CVB3 VP1 constructs that had either N-terminal 56 residues or C-terminal 35 residues of VP1 fused with GST. These segments correspond to the regions outside the globular core of VP1 (Fig. 2a ^9^).

### Western blotting

For Western blotting, concentrated Coxsackievirus B 1-6 stocks were prepared to test the capability of 3A6 in recognizing CVB-group antigens^29^. CVB1-5 originated from field isolates^33^ whereas the CVB6 ATCC strain Schmitt was used. Filtered supernatants from pelleted and lysed CVB1-6-infected cells (0.66 µg/virus) were analyzed and 8 µg of cell lysates were used as negative control. Two parallel analyses were performed on Mini-Protean® TGX Stain-free™ gels (BioRad) and the total protein was imaged using ChemiDoc™ XRS + imaging system (BioRad). The BSA-blocked membranes were stained with 5D8/1 (1:3000) or with 3A6 (1:1000). IRDye®800CW Goat anti-Mouse (Li-Cor) for 5D8/1 and IRDye®680RD Goat anti-Rat for 3A6 were used as secondary antibodies and the staining was detected with Odyssey Clx (Li-Cor).

### Immunocytochemistry, immunohistochemistry and immunofluorescence

The suitability of 3A6 for immunostainings was evaluated using a variety of samples from different origins in two independent laboratories (Tampere, Finland and Exeter, UK) and its performance was compared to 5D8/1. Chromogenic immunocytochemistry (ICC) and immunohistochemistry (IHC) was performed using a standard immunoperoxidase (IHC-P) approach for paraffin sections as previously described^19,24^. Following optimization, antibodies were used at the following concentrations: 3A6 (Tampere 1:150; 8.8 µg/ml; Exeter 1:100; 13.2 µg/ml), 5D8/1 (Tampere 1:100; 700 ng/ml; Exeter 1:1400; 55 ng/ml). For IF, sections were deparaffinized and heat-induced epitope retrieval in 10 mM citrate pH6 was performed. Sections were subsequently blocked with goat serum and primary antibodies were applied for 1 h at RT. For the detection of the primary antibodies, polyclonal goat anti-mouse Alexa Fluor 488 and goat anti-rat Alexa Fluor 488 or 555 (Invitrogen, Grand Island, NY) were applied for 1 h at RT. After washing, sections were mounted with ProLong Diamond antifade with DAPI (Thermo Fisher Scientific). Control slides were processed in the absence of primary antibody or with rat isotype control (BioLegend®) to confirm the specificity of labeling. Coverslip IF for PFA fixed and CVB1-infected PANC1 cells was performed by first permeabilizing the samples with 0.2% Triton X-100D in PBS and then incubating the primary antibodies 3A6 (1:250), or mAb anti-dsRNA (1:500; J2, English & Scientific Consulting, Hungary) at RT for 40 min. Detection was done using the secondary Alexa Fluor antibodies (488 or 555) by incubating at RT for 20 min. Paraffin slides were visualized with an Olympus BX60 microscope fitted with an Olympus Colorview III camera (Tampere) or a Nikon 50i Microscope fitted with a DS-Fi camera and a DSL2 camera control unit (Exeter). PFA-fixed coverslip were imaged with Nikon Eclipse Ti-E A1 + laser scanning confocal microscope system (Tampere).

Paraffin sections from cell microarrays (Laiho *et al*. 2015) were immunostained to study the spectrum of different EV serotypes recognized by clone 3A6. The relative sensitivity of the clone in recognizing CVB1 when compared to other EV antibodies and detection methods (such as RT-PCR, *in situ* hybridization, proteomics^24^) was also tested. PFA-fixed human ductal pancreatic carcinoma (PANC1) cells infected with CVB1 and paraffin sections from CVB-1 and 3 infected mouse pancreata^21^ were used to study the performance of 3A6 in samples with different pretreatments. Paraffin embedded human tissue samples from neonatal heart with confirmed CVB infection as well as pancreas and spleen samples were also tested to evaluate the ability of 3A6 to detect virus in human tissue and to determine if any non-specific binding occured in these samples. For the control and CVB-infected heart tissue either the ImmPRESS HRP Anti-Mouse (5D8/1) or anti-Rat (3A6) IgG (Peroxidase) Polymer Detection Kits were used to visualize the antigens.

### Peptide immunofluorescent assay for 3A6

For mapping of the 3A6 epitope, two VP1 peptides that span the known 5D8/1 epitope^19^ were used. High-binding enzyme immunoassay plates were coated with the peptides (RPTNSESIPALTAAE or PALTAVETGATNPLV; Genscript, Piscataway, USA and previously described in Richardson^19,34^ or with CVB3 VLP^20^ in 50 mM sodium carbonate buffer pH 9.4 overnight. Plates were then blocked with 5% normal goat serum (NGS; Vector) in PBS and incubated for 2 hr with varying dilutions of clone 5D8/1 or 3A6 in 5%NGS/ PBS. The binding of the antibody was detected with Alexa 488-conjugated anti-mouse IgG or anti-rat IgG (Life Technologies; 1/400). Fluorescence was measured using a Pherastar FS plate reader.

### Cryo-immuno electron microscopy

The suitability of 3A6 in cryo-immuno-EM was evaluated in comparison to 5D8/1. Pancreas tissue from NOD mice that had been infected with CVB1 (10^5^ PFU/mouse) was collected 3 days post-infection and labeled using both 5D8/1 and 3A6 antibodies. Tissue sections were fixed in 4% PFA in 0.1 M phosphate buffer at room temperature, continued at 4 °C for 48 hr, then small tissues pieces were immersed in 2.3 M sucrose in PBS at 4 °C for several days, and frozen in liquid nitrogen. Thin cryosections were cut with a Leica Ultracut UCT microtome. Immunolabeling was performed as described by Slot and Geuze^35^. Briefly, antibodies were diluted in 1% BSA, 0.1% gelatin in PBS and gold conjugate (10 nm gold particles labeled with protein A; Cell Microscopy Core, UMC, Utrecth, Netherlands) was diluted in 0.1% BSA in PBS. For the rat 3A6 antibody, diluted rabbit-anti-rat IgG (Bioss) was used as a bridging antibody before protein A gold incubation. Controls were prepared by carrying out the labeling procedure without primary antibody or in non-infected tissue. The sections were embedded in methylcellulose and examined with a Jeol 1400 microscope.

### Virus neutralization assay

CVB1 (V200 strain) was used to analyze the ability of the 14 monoclonal antibodies to neutralize virus infectivity. 100 PFUs of the virus was added to dilutions (from 1/4 to 1/64) of hybridoma culture supernatants in equal volumes (3 µl), and allowed to interact at 37 °C for one hour followed by an overnight incubation at RT. The number of plaques generated by antibody-treated viruses was quantified in GMK cells in 12 well plates. The number of plaques for each virus and each dilution was compared to mock-treated virus and a reduction of plaque number of more than 80% of the untreated virus plaques was considered positive. A horse hyperimmune neutralizimg CVB1 was used as positive control in the assay. Antisera from terminal bleeds from four of the rats were also tested in neutralization assays with same dilutions used for the supernatants.

## Electronic supplementary material

Supplementary
