# Supplementary material for: A novel rat CVB1-VP1 monoclonal antibody 3A6 detects a broad range of enteroviruses
Source: Sci Rep. 2018 Jan 8;8:33. doi: 10.1038/s41598-017-18495-4 (PMC5758616; doi:10.1038/s41598-017-18495-4)
Supplement: Supplementary file 1 — Supplementary [file 41598_2017_18495_MOESM1_ESM.pdf]

A novel rat CVB1-VP1 monoclonal antibody 3A6 detects a broad range of enteroviruses

Niila V. V. Saarinen[1], Jutta E. Laiho[1], Sarah J. Richardson[2], Marie Zeissler[2], Virginia M. Stone[3], Varpu Marjomäki[5], Tino Kantoluoto[5], Marc S. Horwitz[6], Amirbabak Sioofy-Khojine[1], Anni Honkimaa[1], Minna M. Hankaniemi[1], Malin Flodström-Tullberg[3], Heikki Hyöty[1,4], Vesa P. Hytönen[1,4], Olli H. Laitinen[1]

[1] Faculty of Medicine and Life Sciences, University of Tampere, Tampere, Finland

[2] University of Exeter Medical School, Exeter, Devon, UK

[3] Department of Medicine HS, Karolinska Institutet, Stockholm, Sweden

[4] Fimlab Laboratories, Pirkanmaa Hospital District, Tampere, Finland

[5] Department of Biological and Environmental Science / Nanoscience center, University of Jyväskylä, Jyväskylä, Finland

[6] Department of Microbiology and Immunology, Life Sciences Institute, University of British Columbia, Vancouver, Canada

```
CLUSTAL O(1.2.4) multiple sequence alignment

CAV2-Fleetwood                      --ANTQVSQHSIETGRVPALQAAETGATSNASDENLIE   EV-A
CAV4-High-point-AY421762.1          --ANTAPSSHSLNTGLVPALQAAETGASSTATDGNLIE
PV3                                 ALPNTE-ASGPTHSKEIPALTAVETGATNPLVPSDTVQ   EV-C
Cav9-Griggs-D00627.1                --ADTM-RTGPSNSASVPALTAVETGHTSQVTPSDTM-
Echovirus6-FIN09-NPA-               --ADTM-PSGPSNSESIPALTAAETGHTSQVVPSDTI-
CVB1-pMP1.23-AY186746.1             --ADTV-SSRPTNSESIPALTAAETGHTSQVVPSDTM-
CVB3                                --ADTV-GTGPTNSEAIPALTAAETGHTSQVVPGDTM-   EV-B
CVB5-Faulkner-AF114383.1            --ADTI-GSGPVNSESIPALTAAETGHTSQVVPADTM-
Echo3-Morrisey                      --ADTM-PTGPRNTESVPALTAVETGHTSQVVPGDTM-
CVB2-Ohio-1-AF085363.1              --ADTI-GSGPSNSEAIPVLTAVETGHTSQVTPSDTM-
CVB4-E2-AF311939.1                  --ADTI-ARGPSNSEQIPALTAVETGHTSQVDPSDTM-
CVB6-Scmitt-AF105342.1              --ADTM-PSGPTNSEAVPALTAVETGHTSQVVPSDNM-
Echo9-Hill-X84981.1                 --ADTI-RSGPSNSEAVPALTAAETGHTSQVVPSDTM-
                                       :*       .:  :*.* *.*** :.     : :
```

Figure S1. Sequence alignment around the presumed epitope area of 5D8/1(Purple) of selected enteroviruses in the N-terminal of VP1 proteins. Highlighted in red the most obvious difference between PV3 and EVB-viruses at position (40-41) in relation to CVB1 VP1 aminoacid sequence. 3A6 detected PV3 in fluorescent assays, but not in WB, whereas *EV-A* viruses gave no signal in either.

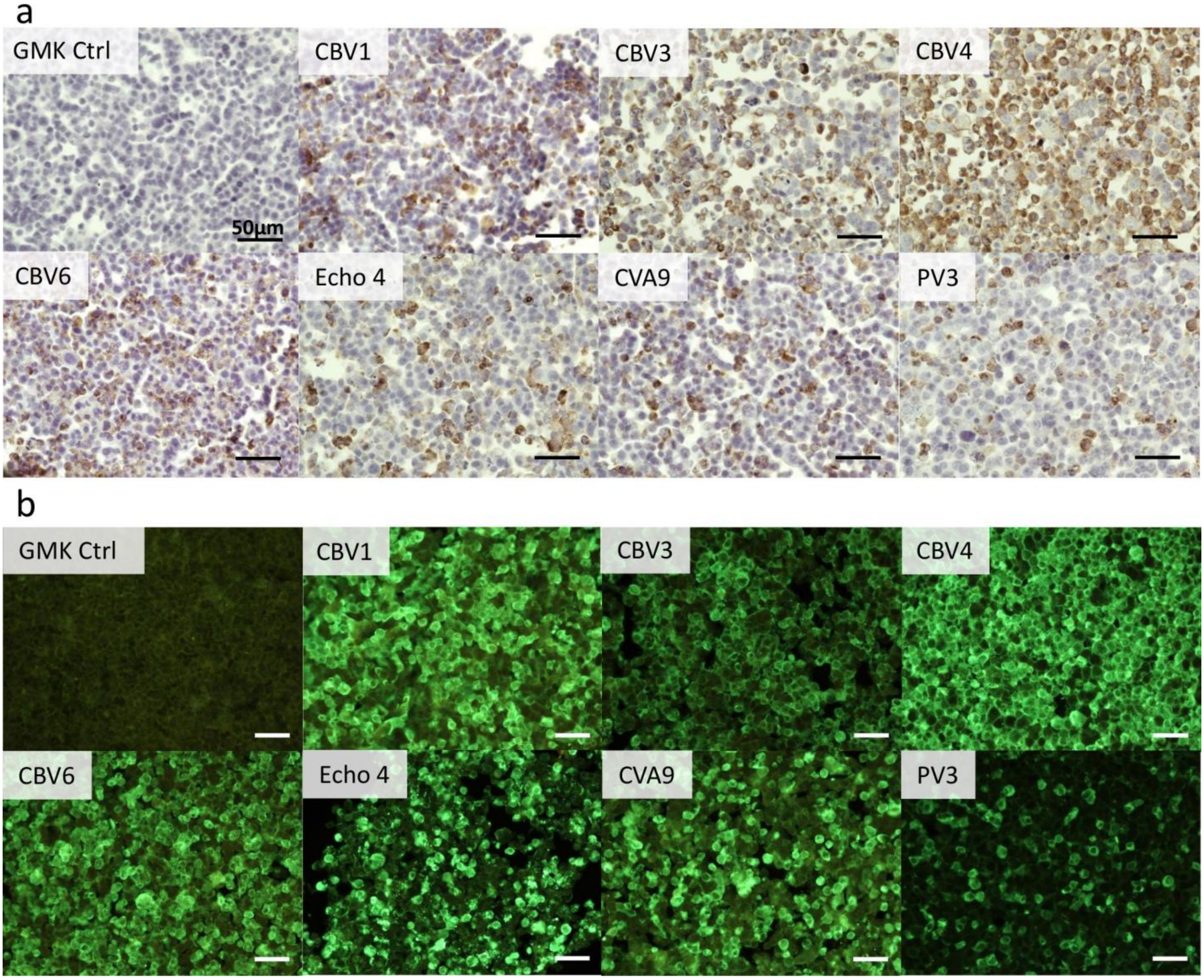

Figure S2. Detection of EV infected cells with 3A6. Representative images of 3A6 IHC-P (panel a) and IF (panel b) staining in GMK control cells and in acutely-infected CVB1,3,4,6, Echo4, CVA9 and PV3 cells.

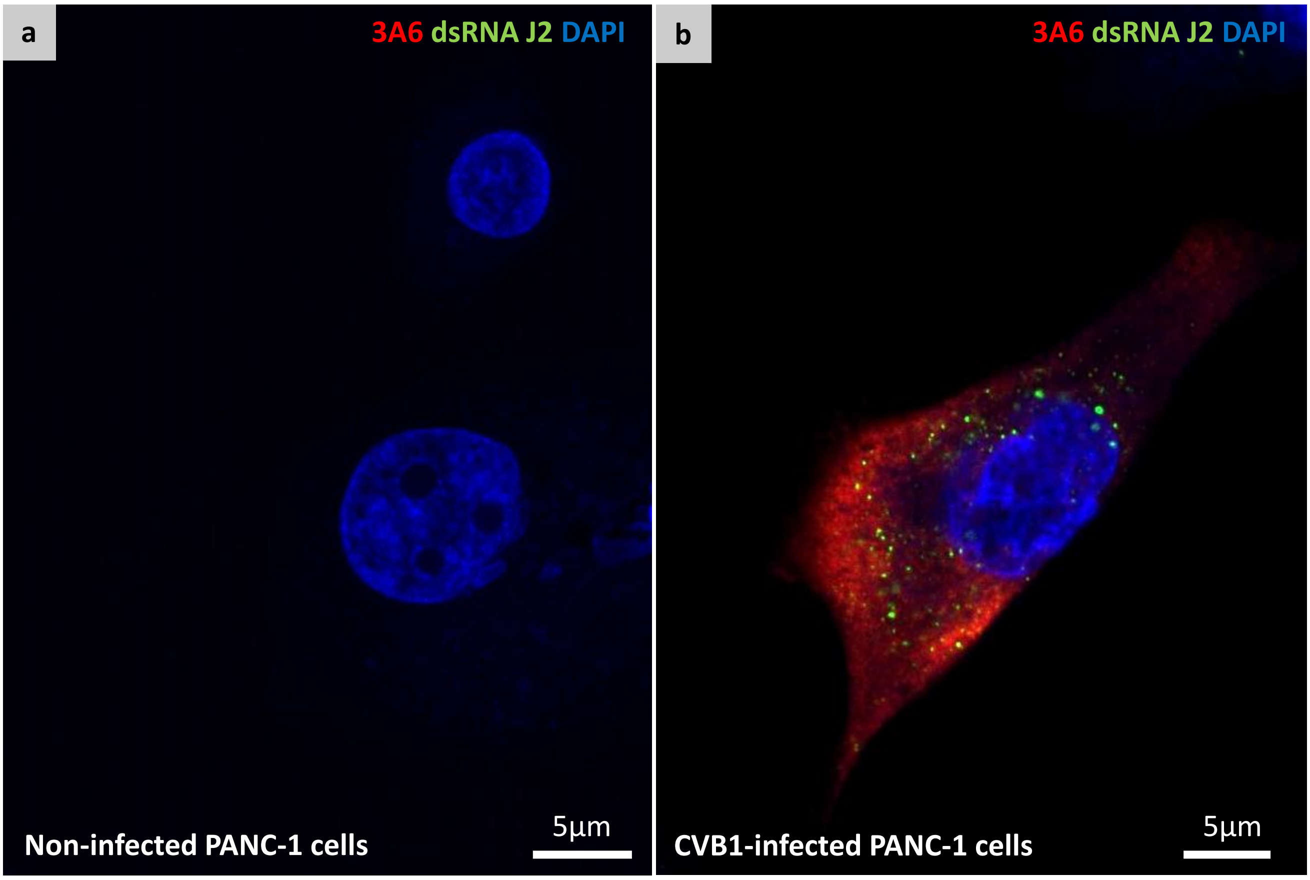

Figure S3. 3A6 localizes to same cells as dsRNA (J2). A merged confocal image of PFA-fixed non-infected control cells a) and CVB1-infected PANC-1 cells b) double stained with 3A6 (red) and dsRNA antibody J2 (green). Cell nuclei were stained with DAPI (blue).

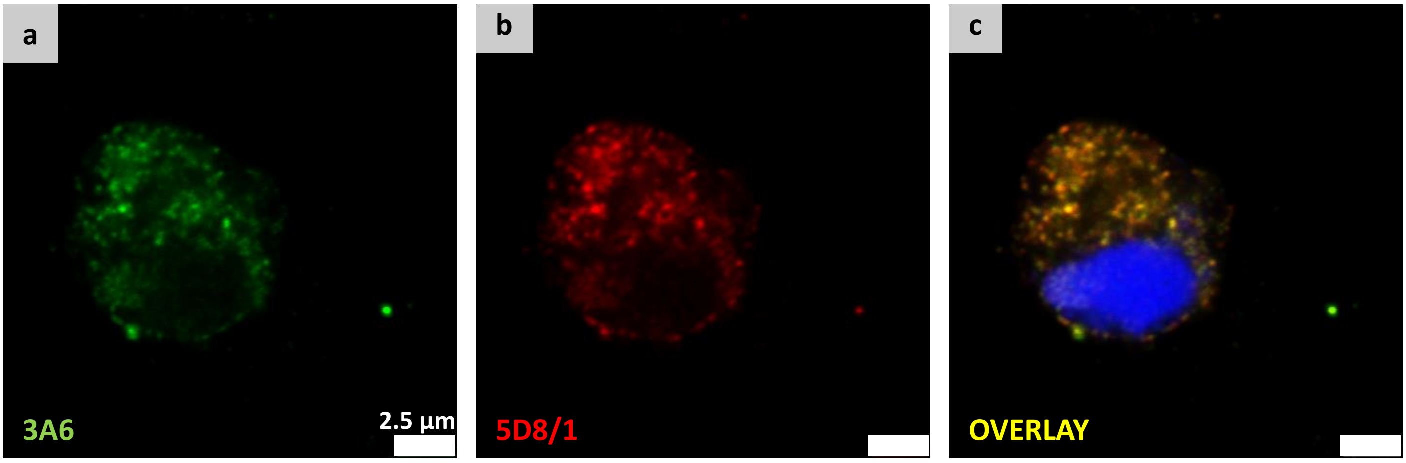

Figure S4. Double staining of infected Vero cells with 3A6 and 5D8/1. High magnification image of a CVB1-infected Vero cells stained with a) 3A6, b) 5D8/1 and c) merged image, demonstrates colocalization of the two antibodies.
